# Growth factor independence 1 expression in myeloma cells enhances their growth, survival, and osteoclastogenesis

**DOI:** 10.1186/s13045-018-0666-5

**Published:** 2018-10-04

**Authors:** Daniela N Petrusca, Denise Toscani, Feng-Ming Wang, Cheolkyu Park, Colin D Crean, Judith L Anderson, Silvia Marino, Khalid S Mohammad, Dan Zhou, Rebecca Silbermann, Quanhong Sun, Noriyoshi Kurihara, Deborah L Galson, Nicola Giuliani, G David Roodman

**Affiliations:** 1 Department of Medicine, Division of Hematology-Oncology, Indiana University School of Medicine, 980 Walnut Street, Walther Hall, Room C346, Indianapolis, IN 46202 USA; 2 Myeloma Unit, Department of Medicine and Surgery, University of Parma, Parma, Italy; 3 Endodontics, Texas A&M University College of Dentistry, Dallas, TX USA; 4 Department of Medicine, Division of Endocrinology, Indiana University School of Medicine, Indianapolis, IN USA; 5 Department of Medicine, Division of Hematology-Oncology, UPMC Hillman Cancer Center, McGowan Institute for Regenerative Medicine, University of Pittsburgh, Pittsburgh, PA USA; 6 Rodebush VA Medical Center, Indianapolis, IN USA

**Keywords:** Gfi1, Multiple myeloma, p53, Bone disease, Apoptosis and osteolysis

## Abstract

**Background:**

In spite of major advances in treatment, multiple myeloma (MM) is currently an incurable malignancy due to the emergence of drug-resistant clones. We previously showed that MM cells upregulate the transcriptional repressor, growth factor independence 1 (Gfi1), in bone marrow stromal cells (BMSCs) that induces prolonged inhibition of osteoblast differentiation. However, the role of Gfi1 in MM cells is unknown.

**Methods:**

Human primary CD138+ and BMSC were purified from normal donors and MM patients’ bone marrow aspirates. Gfi1 knockdown and overexpressing cells were generated by lentiviral-mediated shRNA. Proliferation/apoptosis studies were done by flow cytometry, and protein levels were determined by Western blot and/or immunohistochemistry. An experimental MM mouse model was generated to investigate the effects of MM cells overexpressing Gfi1 on tumor burden and osteolysis in vivo.

**Results:**

We found that Gfi1 expression is increased in patient’s MM cells and MM cell lines and was further increased by co-culture with BMSC, IL-6, and sphingosine-1-phosphate. Modulation of Gfi1 in MM cells had major effects on their survival and growth. Knockdown of *Gfi1* induced apoptosis in p53-wt, p53-mutant, and p53-deficient MM cells, while *Gfi1* overexpression enhanced MM cell growth and protected MM cells from bortezomib-induced cell death. Gfi1 enhanced cell survival of p53-wt MM cells by binding to p53, thereby blocking binding to the promoters of the pro-apoptotic *BAX* and *NOXA* genes. Further, Gfi1-p53 binding could be blocked by HDAC inhibitors. Importantly, inoculation of MM cells overexpressing Gfi1 in mice induced increased bone destruction, increased osteoclast number and size, and enhanced tumor growth.

**Conclusions:**

These results support that Gfi1 plays a key role in MM tumor growth, survival, and bone destruction and contributes to bortezomib resistance, suggesting that Gfi1 may be a novel therapeutic target for MM.

**Electronic supplementary material:**

The online version of this article (10.1186/s13045-018-0666-5) contains supplementary material, which is available to authorized users.

## Background

Multiple myeloma (MM) is characterized by uncontrolled growth and accumulation of malignant plasma cells in the bone marrow (BM) [1]. Bone destruction is a hallmark of MM, occurring in over 80% of patients and severely impacting patients’ quality of life [2]. Modern treatment approaches have markedly improved MM patient survival, but MM remains incurable for most patients [3, 4] due to the emergence of drug-resistant clones. Thus, novel treatments are needed if we are to cure MM.

We recently reported that MM cells upregulate the transcriptional repressor growth factor independence 1 (Gfi1) in bone marrow stromal cell (BMSC), which induces epigenetic changes in the *Runx2* gene to inhibit osteoblast (OB) differentiation [5] thereby increasing MM cell growth and chemoresistance [5]. Gfi1 encodes a nuclear zinc finger DNA-binding protein that also acts as a transcriptional repressor of genes involved in hematopoiesis and hematopoietic stem cell self-renewal and quiescence [6]. It recruits the histone demethylase complex LSD-1/CoRest and the histone deacetylases HDAC-1, HDAC-2, and HDAC-3 to promoters of specific target genes to reversibly repress transcriptional activity [7, 8]. Gfi1 overexpression in normal T cells delays apoptosis, thereby protects them from growth factor withdrawal [9–11], as well as enhances the progression of murine T cell acute leukemia (T-ALL) [12]. Further, Gfi1 cooperates with oncoproteins, such as Myc and Pim-1, to induce development of lymphoma and ALL [13]. Gfi1 protein levels are differentially regulated by the ubiquitin-proteasome system during myeloid differentiation with rapid proteasomal degradation in granulocytes and stabilization in immature myeloid cells [14].

Gfi1 can also interact with the p53 tumor suppressor [15, 16]. Du et al. showed that p53 binds the Gfi1 core promoter to repress Gfi1 transcription, and Gfi1 inhibits DNA damage-induced apoptosis in hematopoietic cells [17]. Downregulation of p53 increases Gfi1 expression while reactivation of p53 reduces Gfi1 expression. Further, Gfi1 overexpression inhibits apoptosis while Gfi1 knockdown increases cell death induced by DNA damage, suggesting that p53 may induce apoptosis through downregulation of Gfi1 [17]. Finally, Gfi1 also decreases the pro-apoptotic effects of p53 in lymphoblastic leukemia by binding to the regulatory regions of pro-apoptotic genes, such as *BAX*, *Pmaip1* (*NOXA*), and *Bbc3* (*PUMA*); blocking p53 binding; and decreasing methylation of p53-K372 [12].

Because Gfi1 can play an important role in other lymphoid malignancies [12], we determined if Gfi1 also contributes to MM cell growth, survival, and chemoresistance. We report that Gfi1 mediates MM cell growth and viability, enhances MM cell resistance to bortezomib-induced cell death in vitro, and increases MM cell growth and osteoclastogenesis in vivo.

## Methods

### Human primary CD138+ and BMSC cell purification and MM cell lines

Bone marrow (BM) aspirates were collected in heparin from 9 healthy subjects and 36 patients with plasma cell disorders (Additional file 1 Tables S1 and S2). Non-adherent marrow mononuclear cells were collected, and highly purified MM cells (> 90%) were isolated by magnetic cell fractionation with anti-CD138 MicroBeads (Miltenyi Biotec Inc., San Diego, CA) [18] as previously described [19, 20]. The remaining adherent cells were cultured for 21 days with media changes every 4 days to obtain BMSCs that were used at passages 2 and 3.

Myeloma cell lines were purchased from ATCC (Manassas, VA) (MM.1S; H929) and from Leibniz-Institut DSMZ (Braunschweig, Germany) (MOLP-8) or generously provided by Drs. Louis Stancato (U266), Kenneth Anderson (KMS-11), and Nicola Giuliani (JJN3). HEK293-T cell line was purchased from ATCC (Manassas, VA), and SAKA-T cell line was generated by our group [21].

### Cell viability/proliferation assays

#### Cell viability assays

Human MM cell lines were incubated in 96-well plates in RPMI-1640 media with 10% FCS and varying concentrations of Btz for 24 and 48 h. Cell’s viability was quantified using MTT assays (Sigma-Aldrich, St. Louis, MO) or alamarBlue® Cell Viability assays (Thermos Fisher Scientific, Waltham, MA), per the manufacturer’s protocol.

#### Proliferation assays

Single cell suspensions of MM cells were stained with 1 μM CellTrace for 20 min at 37 °C according to the manufacturer’s protocol and grown in complete medium for 72 h. Fresh stained cells were used as controls.

#### Cell cycle/apoptosis assays

Propidium iodide (PI) staining was used to detect cell cycle phases, and a fluorescein labeling system was employed to detect dUTP end nicks according to the manufacturer’s instructions (APO-BRDU kit; BD Bioscience, San Jose, CA) using flow cytometry (Fortessa flow cytometer, Becton Dickinson). Post-acquisition analysis of the gated cell subsets was performed using FlowJo software (Tree Star, OR).

### In vivo studies

Fox Chase Beige SCID female mice (4–6 weeks of age) (Charles Rivers, Indianapolis, IN) were inoculated intratibially (IT) with 10^5^ MM.1S cells stably transduced with empty vector (EV) or overexpressing *Gfi1* (Gfi1 o/e) in 20 μl of PBS. Mice were maintained and handled in accordance with the Guide for the Care and Use of Laboratory Animals on a protocol approved by the Indiana University IACUC. The animals were followed for 8 weeks before euthanasia due to large tumor development. X-ray images of dissected tibias were acquired on a viva CT 40 scanner (Scanco Medical) at a resolution of 21 m isotropic, reconstructed, and segmented for 3D display using the instruments analysis algorithm software (Sanco Medical Evaluation Program V6).

### Ig lambda concentrations

Human plasma Igλ concentrations were measured after 5 weeks post-IT injection to define successful myeloma engraftment and at the end of the study to evaluate tumor burden using commercially available ELISA kits, according to manufacturer’s instructions (Bethyl Laboratories, Inc., Montgomery, TX, USA).

### Histology and histomorphometry

The hind limbs were fixed with formalin and decalcified in 10% EDTA for 2 weeks. The tissues were processed as previously described [22], and the sections stained with hematoxylin and eosin, and tartrate-resistant alkaline phosphatase (TRAP) (Sigma-Aldrich, St. Louis, MO, USA). The sections were scored on a Leica DM LB compound microscope outfitted with a Q-Imaging Micropublisher Cooled CCD color digital camera (W. Nuhsbaum Inc., McHenry, IL, USA).

### Osteoclast cultures

Mouse bone marrow cells were flushed from the long bones of 3–5-month-old mice, and non-adherent cells were collected and incubated in αMEM supplemented with M-CSF (10 ng/ml) for 48–72 h to generate bone marrow monocytes as previously described [23]. For myeloma-osteoclast co-cultures, M-CSF-generated bone marrow monocytes were plated into 96-well culture plates (1 × 10^5^ cells/well) in αMEM supplemented with RANKL (50 ng/ml) and MM.1S EV, and MM.1S Gfi1 o/e myeloma cells (5000 cells/well) were added 6 h later. The cultures were continued for 4 days and then fixed in 4% paraformaldehyde, washed with PBS, and stained for TRAP using a leucocyte acid phosphatase staining kit (Sigma-Aldrich, St. Louis, MO, USA). TRAP-positive cells with three or more nuclei were scored as osteoclasts by counting with an Olympus CKX41 inverted microscope using a × 10 objective.

### Statistical analyses

Statistical analyses were performed using Prism software (Irvine, CA). The differences between groups were compared using a two-tailed unpaired Student *t* test or ANOVA. Statistically significant difference was set at *p* < 0.05, and results are expressed as mean ± SEM. Representative data from at least three biologic replicates are shown.

The specific details for the other experimental methods and procedures employed (chemicals and antibodies; plasmid and lentiviral constructs; immunoprecipitation and Western blotting; real-time RT-PCR (qPCR); ChIP assay and immunofluorescence) are listed in Additional file 2 (available on the journal website).

## Results

### Gfi1 expression is upregulated in human MM cells

**Fig. 1:**
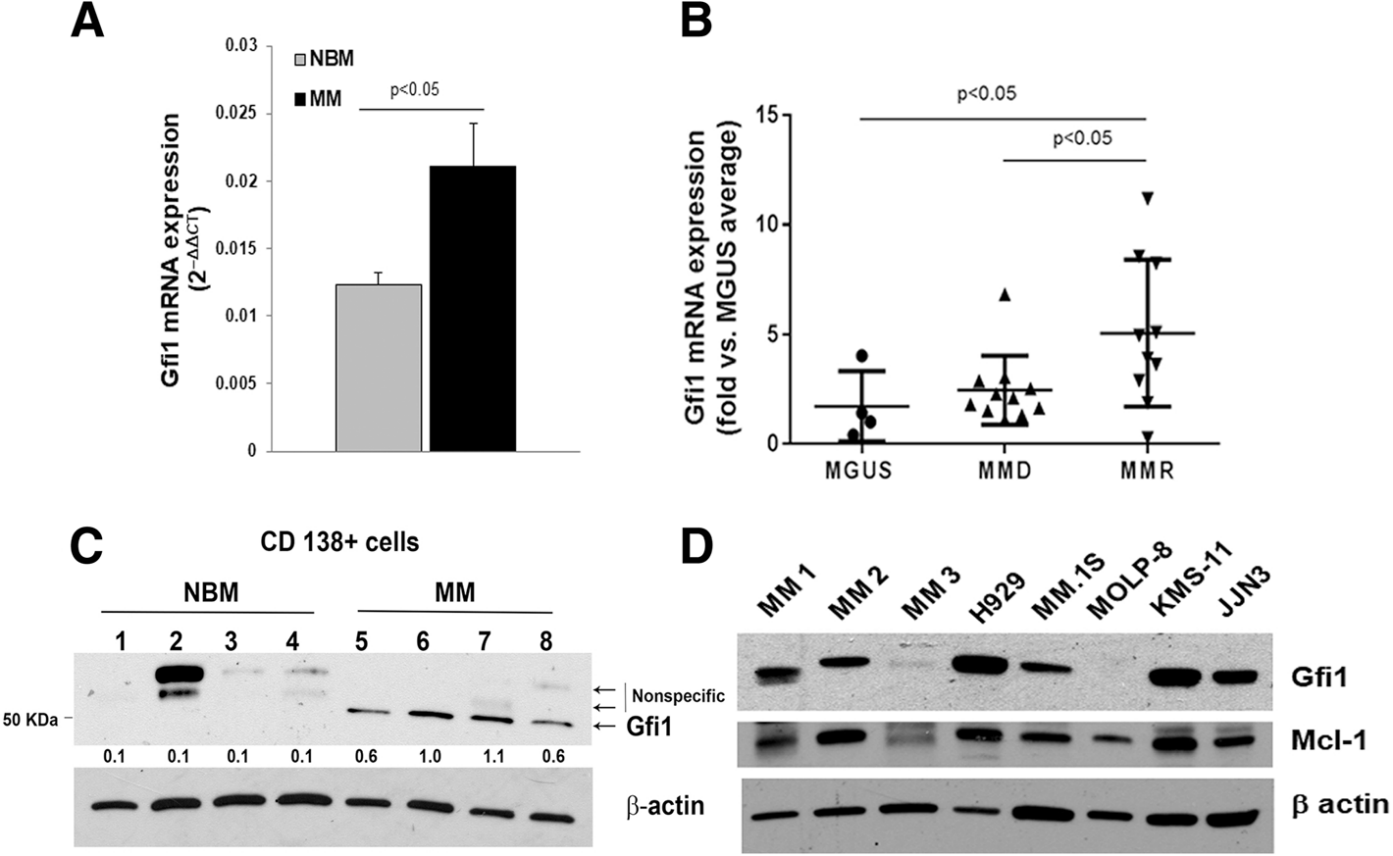
Gfi1 expression is upregulated in human MM cells. Gfi1 mRNA expression was measured by qPCR in CD138+ cells from multiple myeloma patients (MM) (*n* = 7) and normal bone marrow (NBM) donors (*n* = 3) (see patient/normal donor characteristics in Additional file 1: Table S1) (**a**). Gfi1 mRNA expression was measured by qPCR in CD138+ cells from relapsed MM patients (MMR) (*n* = 10), MGUS patients (*n* = 4), and MM newly diagnosed patients (MMD) (*n* = 11) (see characteristics of patients from Italy in Additional file 1: Table S2. Unpaired *t* test with Welch’s correction: MGUS vs MMR *p = 0.0281 and MMD vs MMR *p = 0.0438 (**b**). Gfi1 protein levels were analyzed by WB in cell lysates of primary CD138+ cells isolated from MM patients and normal bone marrow (NBM) donors (numbers represent Gfi1/β-actin ratio of densitometric measurements; *p* < 0.005 MM vs. NBM) (**c**). Gfi1 and Mcl-1 protein levels were analyzed by WB in cell lysates of primary CD138+ cells from MM patients (MM1-3) and MM cell lines using β-actin as loading control (**d**)

*Gfi1* mRNA levels were significantly increased in human CD138+ cells from MM patients compared with normal donors (Additional file 1: Table S1) (Fig. 1a). We then compared *Gfi1* mRNA levels (Additional file 1: Table S2) in different stages of the disease and found that they were increased in relapsed MM patients compared with MGUS patients and newly diagnosed MM patients (Fig. 1b) suggesting that Gfi1 levels correlate with disease progression. Gfi1 protein levels were also significantly higher in CD138+ cells from MM patients and MM cell lines compared with normal donors (Fig. 1c, d).

### Gfi1 mediates the viability and cell growth of myeloma cells

**Fig. 2:**
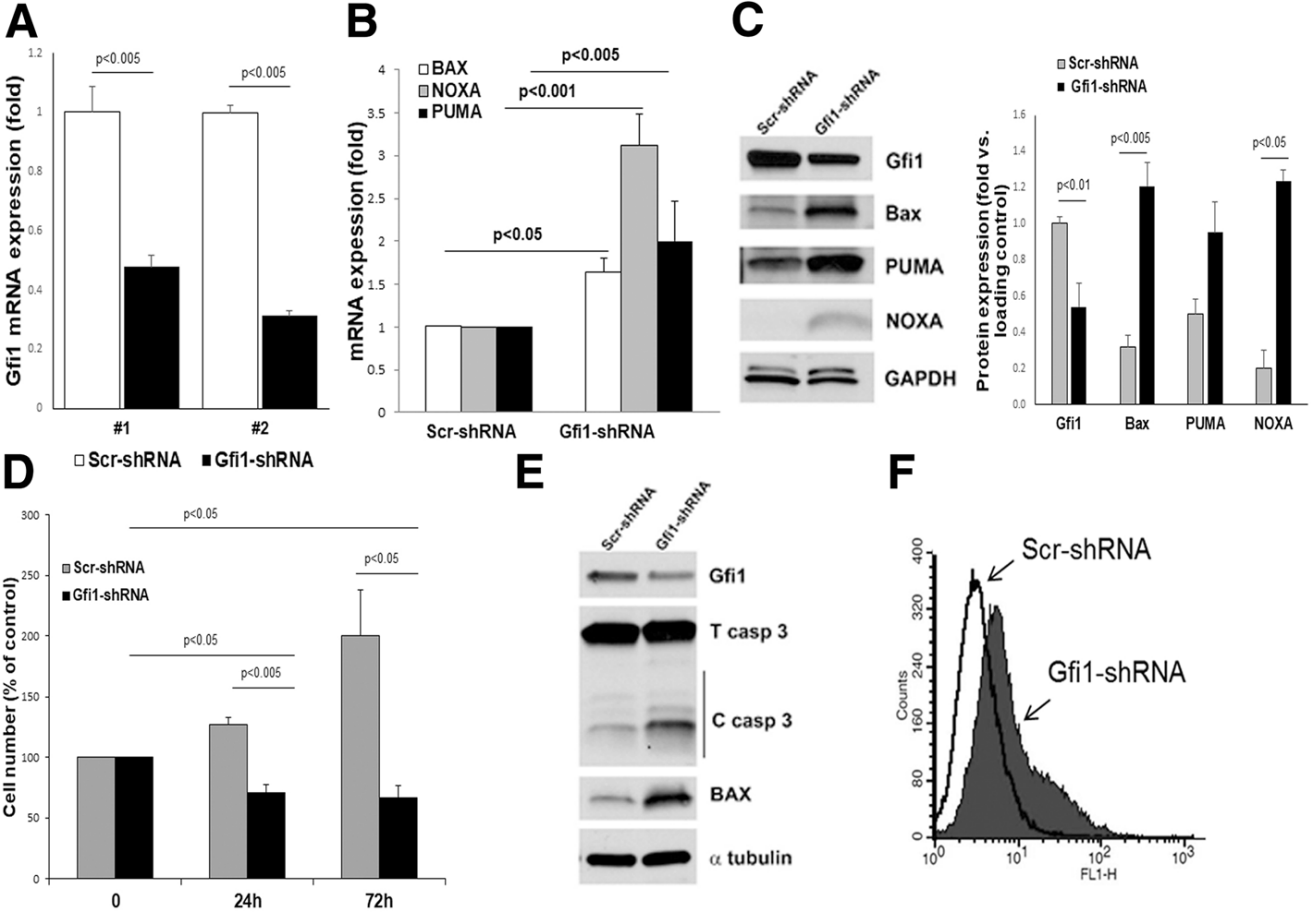
Gfi1 mediates cell growth and viability of myeloma cells. mRNA *Gfi1* levels measured by qPCR showing its knockdown (KD) as compared with scrambled (Scr) control in H929 cells using two different lentiviral shRNA (#1 and #2) (**a**). p53 target genes (*BAX*, *NOXA*, *PUMA*) mRNA levels were measured by qPCR in *Gfi1-KD* and Scr control H929 cells. The bar graph represents fold versus Scr levels (**b**). Gfi1, BAX, NOXA, and PUMA protein levels were analyzed by WB using GAPDH as loading control (left panel). The graph (right panel) represents densitometric levels of the protein versus loading control in three independent experiments (**c**). H929 cells were transduced with *Gfi1-*shRNA (#1 and #2) and Scr-shRNA, selected with puromycin for 48 h and maintained in complete media for another 24 h. The cell number was counted by hemocytometer (time zero) and after another 24 and 72 h. The bar graph represents percent cell number vs time zero (**d**). Gfi1, BAX, total and cleaved caspase 3 protein levels were analyzed by WB using α-tubulin as loading control (**e**) and DNA fragmentation was measured by Apo-BRDU assay and flow cytometry in H929 cells transduced with *Gfi1-*shRNA (#1) and Scr-shRNA (**f**)

We next examined the effects of modulating Gfi1 levels in human MM cell lines that expressed wild-type (wt), mutant, or haploinsufficient p53. Knockdown (KD) of *Gfi1*, using two different shRNA (#1 and #2) (Fig. 2a), significantly increased the expression of the Bcl-2 family pro-apoptotic genes *BAX*, *PUMA*, and *NOXA* (Fig. 2b, c) in H929 cells (p53-wt) and significantly decreased cell growth and viability after 24 and 72 h, compared to scrambled shRNA-transduced control cells (Fig. 2d). The decreased viability of *Gfi1-*KD in MM cells (shRNA#1) resulted from enhanced apoptosis, as shown by increased caspase 3 activation and DNA fragmentation (Fig. 2e, f) and increased levels of sub-G0 cell cycle fraction when compared to scrambled control shRNA (Additional file 3: Figure S1A). Importantly, *Gfi1*-KD (shRNA#1) also induced cell death in MM cells with altered p53. *Gfi1-*KD increased cleaved Mcl-1 and caspase 3 levels in JJN3 (p53-haploinsufficient) and RPMI-8266 (p53-mutant) MM cells (Additional file 3: Figure S1B).

**Fig. 3:**
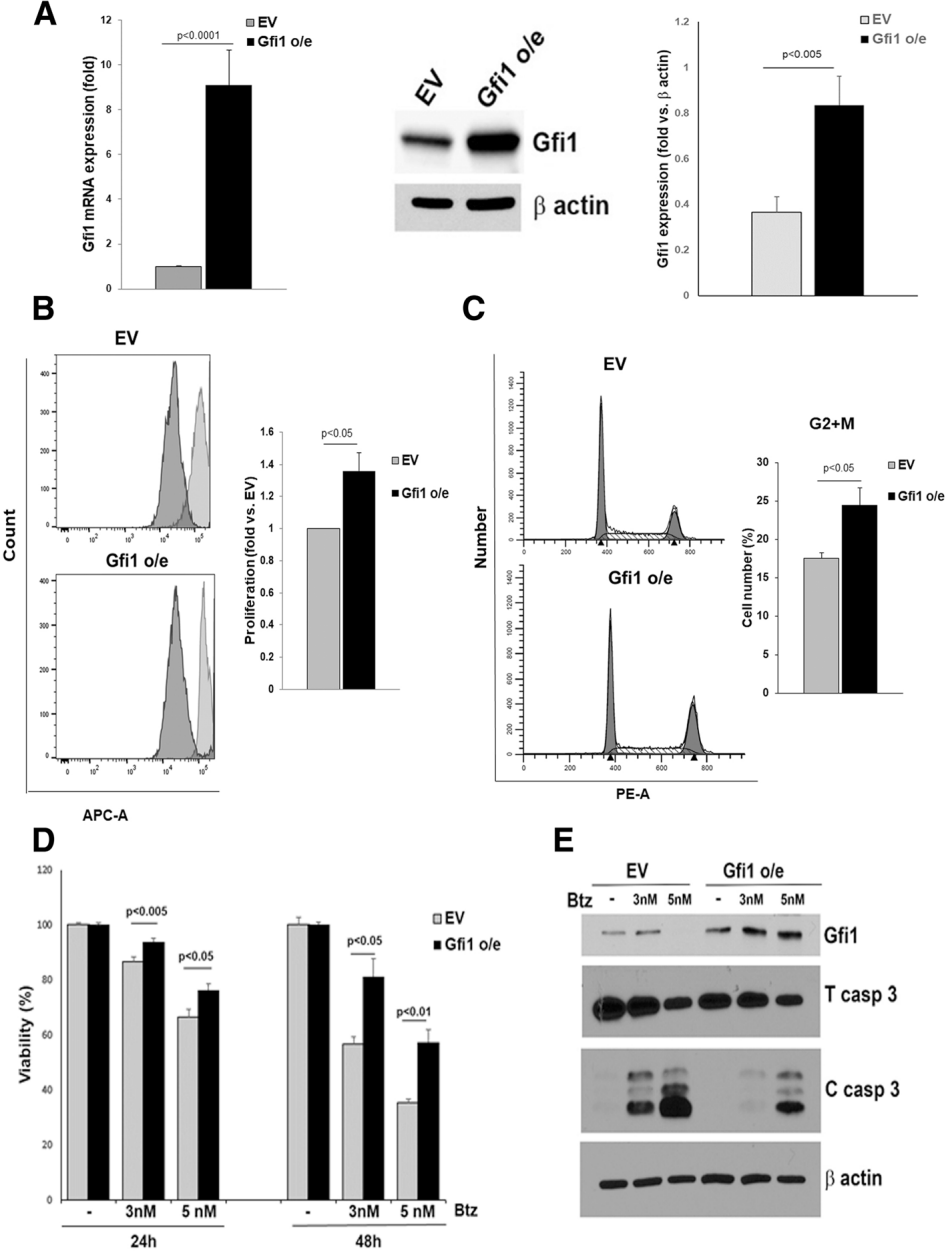
Gfi1 overexpression enhances cell proliferation and survival and protects MM cells from Btz-induced apoptosis. MM.1S cells were transduced with lentivirus carrying the PCL6-GFP vector containing the Gfi1 cDNA insert (Gfi1 o/e) or the empty vector (EV). Gfi1 overexpression was estimated at both mRNA as measured by qPCR (**a**, left panel) and protein levels as measured by WB (**a**, middle panel) and quantified (*N* = 9) by densitometry (**a**, left panel). Stable Gfi1 o/e MM.1S cells and their EV controls were stained with CellTracker, and proliferation was measured after 72 h by flow cytometry (**b**, histograms). The bar graph represents the fold increase in proliferation of Gfi1 o/e cells as compared with EV in three independent experiments (**b**). Cultures of MM.1S cells, Gfi1 o/e, and EV controls (24 h) were stained with propidium iodide (PI), and cell cycle phases were evaluated by flow cytometry (**c**, histograms). The bar graph represents the percent of cells in the “G2+M” cell cycle phase of Gfi1 o/e cells compared with EV in three independent experiments (**c**). MM.1S Gfi1 o/e and EV control cells were treated with Btz at the indicated concentrations. Viability was measured by alamarBlue assay after 24 and 48 h and analyzed as percent from the untreated control (**d**). MM.1S EV and Gfi1 o/e cells were treated for 24 h with Btz (3 and 5 nM), and cell lysates were analyzed by WB for Gfi1 and total and cleaved caspase 3 protein levels using β actin as loading control (**e**)

We then determined the effects of overexpression (o/e) of *Gfi1* in MM cells (Fig. 3a). MM.1S *Gfi1* o/e enhanced proliferation of MM cells compared with MM.1S cells transduced with empty vector (EV), as shown by a significantly decreased CellTrace staining after 72 h (Fig. 3b). Consistent with these results, MM.1S *Gfi1* o/e cells displayed enhanced mitosis with a significantly increased percentage of cells in “G2+M” phase compared with MM.1S EV cells (Fig. 3c), while “S” phase levels were only slightly higher in these cells. The enhanced viability of MM cells overexpressing *Gfi1* was not restricted to p53-wt cells. MTT assays showed that *Gfi1* overexpression in JJN3 cells increased their metabolic activity (Additional file 4: Figure S2A). Importantly, *Gfi1* o/e partially protected MM cells from bortezomib (Btz)-induced apoptosis. Btz treatment dose-dependently decreased MM.1S EV cell viability after 24 and 48 h (Fig. 3d), while *Gfi1* o/e significantly enhanced MM.1S viability (Fig. 3d) and significantly decreased caspase 3 activation (Fig. 3e). This effect was also observed in *Gfi1* o/e JJN3 cells, which had significantly enhanced viability when exposed to Btz compared with EV controls (Additional file 4: Figure S2B). However, *Gfi1* o/e did not protect MM cells from treatment with dexamethasone (data not shown). These results suggest that Gfi1 plays a key role in MM cell survival and contributes to proteasome inhibitor resistance.

### Role of Gfi1-p53 binding in MM cell survival

**Fig. 4:**
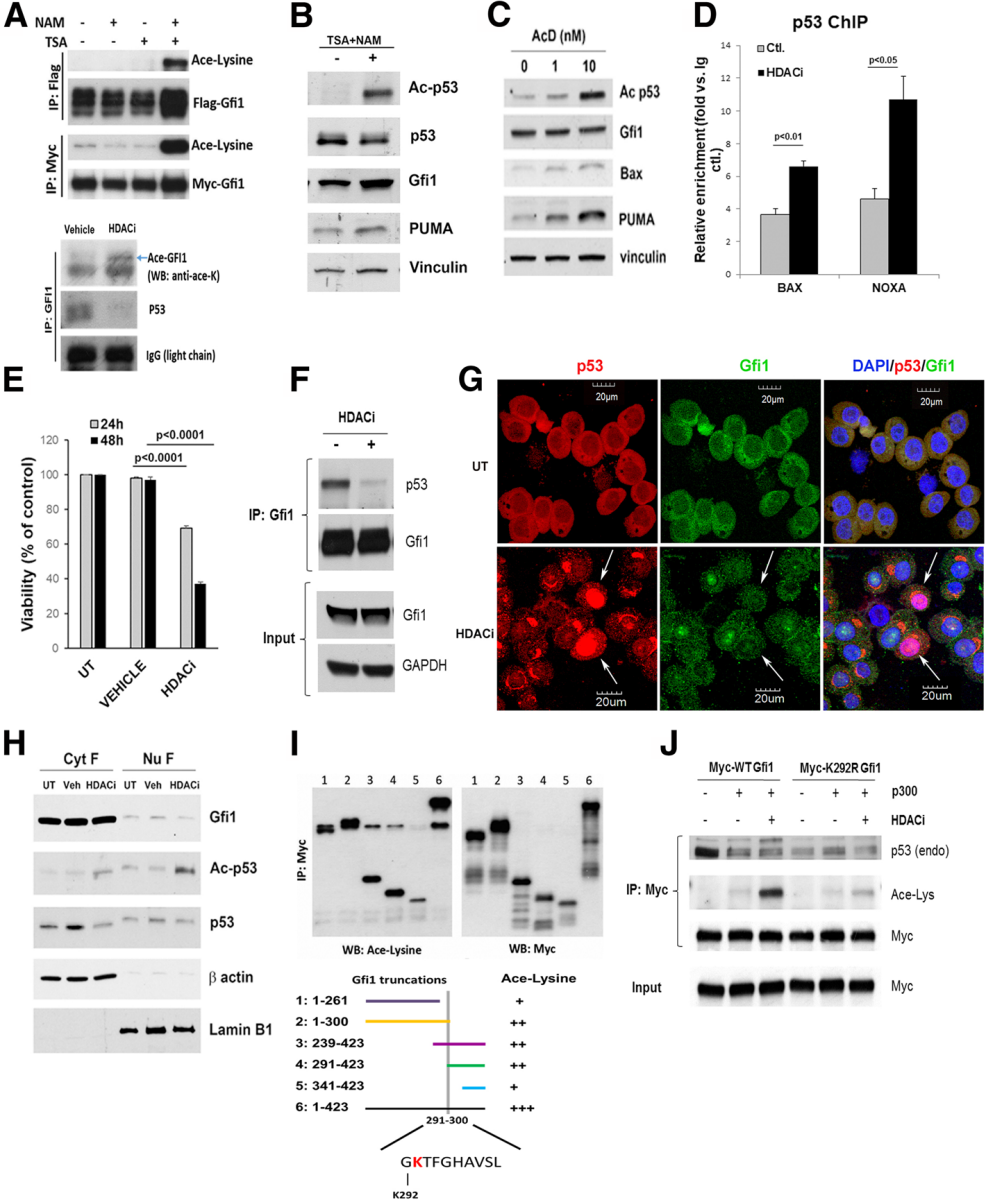
Gfi1 is a target for acetylation and binds p53 in MM cells to prevent apoptosis. HEK293-T cells were transfected with either Flag- or Myc-Gfi1 and HA-P300, incubated 8 h with 10 mM NAM and/or 5 μM TSA (HDACi), and cell lysates were immunoprecipitated (IP) using either an anti-Flag or anti-Myc antibody and analyzed by Western blot (WB) (**a**, top). MM.1S cells, treated with HDACi (8 h), were IP with Gfi1 antibodies and analyzed by WB (**a**, bottom). H929 cells were treated with HDACi (8 h) (**b**) or with actinomycin D (1 and 10 nM; 16 h) (**c**) and analyzed by WB. Chromatin from H929 cells was analyzed using SimpleChIP ® kit and qPCR with ChIP-qPCR primers for BAX and NOXA. The bar graph represents the fold enrichment of p53 protein at *BAX* and *PUMA* promoters (**d**). H929 cell’s viability was measured by MTT assay after 24 and 48 h treatment with HDACi or vehicle control (**e**). H929 cells were treated with HDACi (8 h), IP with anti-Gfi1 antibody, and analyzed by WB (**f**). Paraformaldehyde-fixed cytospins of H929 cells (HDACi for 8 h) were stained for p53 (red-Alexa Fluor® 594), Gfi1 (green-Alexa Fluor® 488), and DAPI for nuclei (Magnification × 40, bar 30 mm). The images are representative of three independent experiments (**g**). H929 cell’s (HDACi; 8 h) cytosolic and nuclear fraction’s (Cell Fractionation kit-Abcam, Cambridge, MA, US) lysates were analyzed by WB with anti-β actin and Lamin B1 used as fraction specific loading controls (**h**). For identifying the acetylation regions in Gfi1, HEK293-T cells were transfected with different forms of Myc-Gfi1 (aa1-261, aa1-300, aa239-423, aa291-423, aa341-423, and aa1-423) and HA-P300, incubated with HDACi (4 h), IP with anti-Myc antibody, and analyzed by WB (**i**). HEK293-T cells were transfected with Myc-Gfi1 (wt) or the Myc-Gfi1 K292R mutant with or without the HA-P300 plasmid, treated with HDACi (8 h), and cell lysates were IP with anti-Myc antibody and analyzed by WB (**j**)

Since Gfi1 binds p53 [12] and KD of *Gfi1* in p53-wt MM cells increased expression of Bcl-2 family proteins, we examined the contribution of p53-Gfi1 binding to the survival of p53-replete MM cells. Gfi1 acts as an epigenetic regulator of gene transcription by recruiting histone deacetylases (HDACs) to promoters of target genes [24]. Since both Gfi1 and p53 undergo post-translational modification, with p53 acetylation activating p53-mediated transcription [25, 26], we determined the role of Gfi1 and p53 acetylation in MM cell survival. Treatment of HEK293-T cells, transfected with plasmids carrying the mouse *Gfi1* cDNA, with TSA (Zn^2+^-dependent HDAC inhibitor (HDACi)) and NAM (NAD+-dependent HDACi) revealed acetylation of the lysine residues of Gfi1 (Fig. 4a, top). TSA plus NAM treatment also resulted in increased acetylated Gfi1 in MM.1S myeloma cells (Fig. 4a, bottom). Moreover, this treatment induced high levels of p53 acetylation in H929 cells (Fig. 4b), as did nanomolar concentrations of actinomycin D (Fig. 4c), a known inducer of p53 acetylation at low doses [27]. p53 acetylation also increased the levels of p53 target proteins (BAX, PUMA) (Fig. 4b, c). Because p53 acetylation increased p53 activity, we assessed the ability of p53 to bind to the *NOXA* and *BAX* promoters. Treatment with HDACi significantly increased the relative enrichment of p53 at these promoters (Fig. 4d).

Since both Gfi1 and p53 can be acetylated in MM cells and their acetylation induced activation of pro-apoptotic genes and decreased viability (Fig. 4d, e), we next assessed if acetylated Gfi1 binds p53. HDACi treatment markedly decreased p53 binding to Gfi1 in MM.1S (Fig. 4a, bottom) and H929 cells (Fig. 4f). Further, immunofluorescence and cell fractionation studies showed that HDACi treatment shifts their cellular distribution. Gfi1 and p53 were co-localized in the cytosol of H929 cells (Fig. 4g), with increased amounts of these proteins in the cytosolic versus nuclear fractions (Fig. 4h). HDACi treatment increased acetylated p53 levels in the nucleus but did not increase nuclear Gfi1 (Fig. 4g, h).

To determine if acetylation of Gfi1, p53, or both was responsible for the enhanced apoptosis seen with HDACi treatment, we analyzed a set of Myc-tagged mGfi truncation constructs for the presence of acetylated lysines. We found several contained acetylated lysines (Fig. 4i), with the most intense bands in an overlapping region from amino acid 291 to 300 that contained K292 in the second zinc finger. We then mutated lysine 292 to arginine (K292R) of Gfi1 and transfected wt Gfi1 and the K292R mutant into HEK293-T cells. Wt Gfi1 co-immunoprecipitated with endogenous p53, but this binding was lost when the cells were exposed to acetylating conditions that strongly acetylated the lysine residues (Fig. 4j). Transfection of HEK293-T cells with the Gfi1 K292R mutant (which will block acetylation of the critical K292 residue in Gfi1, but should not affect p53 acetylation) resulted in decreased p53 binding in untreated cells, but no further loss of p53 interaction was observed in the presence of acetylating conditions (Fig. 4j). This result may suggest that Gfi1-K292 is part of the interaction region for p53, which would also explain why acetylation of K292 could make the complex dissociate. These results demonstrate that Gfi1 acetylation decreases Gfi1 interaction with p53 in human MM cells and that Gfi1-p53 binding prevents p53 binding to the promoters of its pro-apoptotic target genes. Therefore, the acetylation status of Gfi1 appears to be a determining factor contributing to Gfi1’s effects on MM cell survival.

### Microenvironmental factors regulate Gfi1 levels in MM cells

**Fig. 5:**
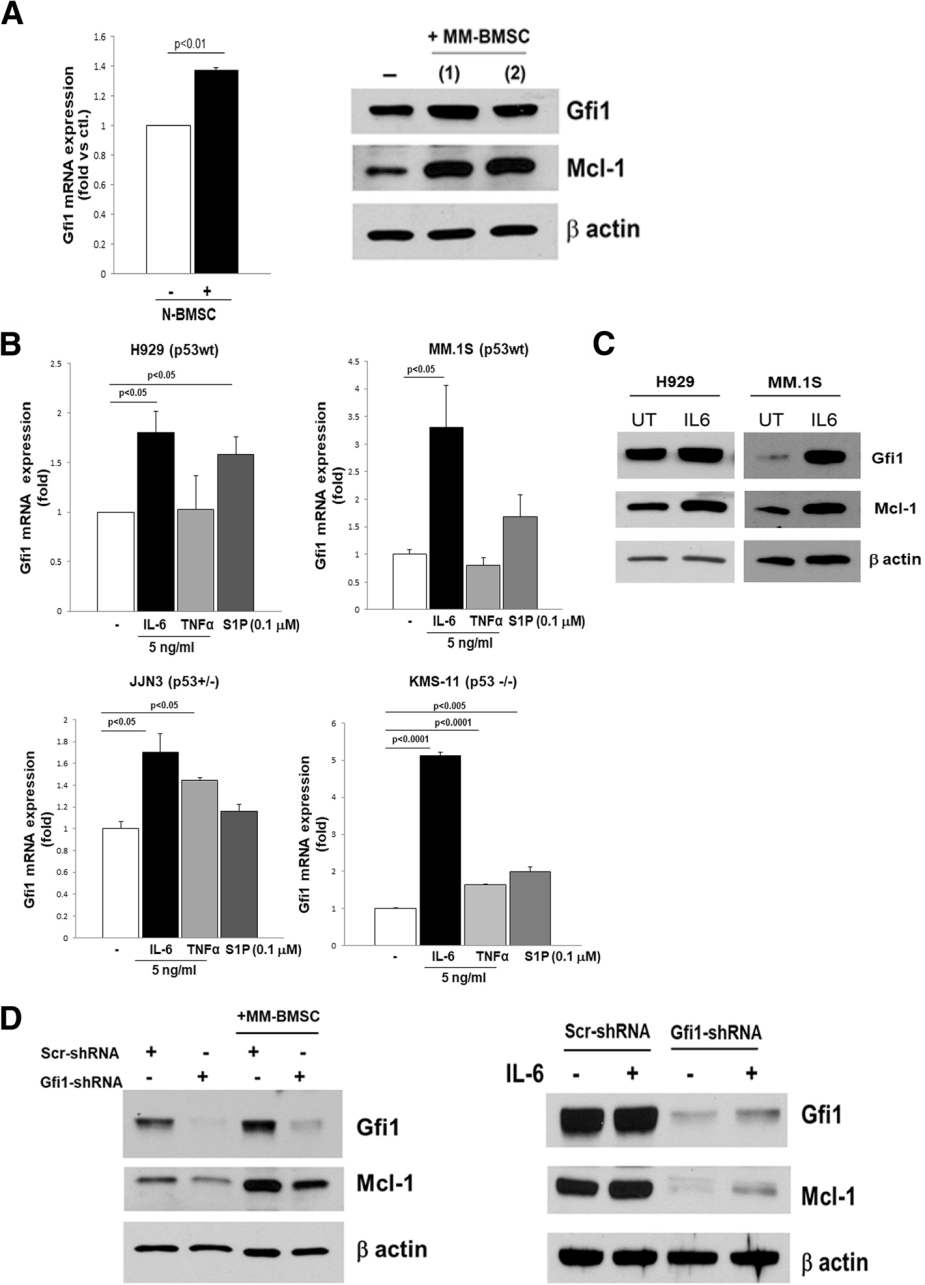
Microenvironmental factors increase Gfi1 levels in MM cells. MM cells (H929) were direct co-cultured for 24 h with marrow stromal cells (BMSCs) obtained from normal donors. MM cells were harvested separate, and *Gfi1* mRNA levels were measured by qPCR and compared with levels in MM cells alone (*N* = 4) (**a**, left panel). H929 cell lysates were analyzed by WB after 24 h direct co-culture with patient BMSC (MM-BMSC) using anti-Gfi1 and Mcl-1 antibodies and β actin as a loading control (**a**, right panel). MM cells with different p53 status: wt (H929 and MM.1S); haploinsufficient (JJN3) and null (KMS-11) were treated for 4 h with IL6, TNFα, and S1P at the indicated concentrations. The bar graph represents *Gfi1* mRNA levels detected by real-time PCR and expressed as fold change versus untreated cells (**b**). H929 and MM.1S cells were treated with and without IL6 (5 ng/ml) for 4 h. Gfi1 and Mcl-1 protein levels in the cell lysates were analyzed by WB using β actin as a loading control (**c**). *Gfi1*-KD and Scr control H929 cells were direct co-cultured for 24 h with patient BMSC (MM-BMSC) or treated for 4 h with IL-6 (5 ng/ml). Gfi1 and Mcl-1 protein levels in the cell lysates were analyzed by WB using β actin as a loading control (**d**)

**Fig. 6:**
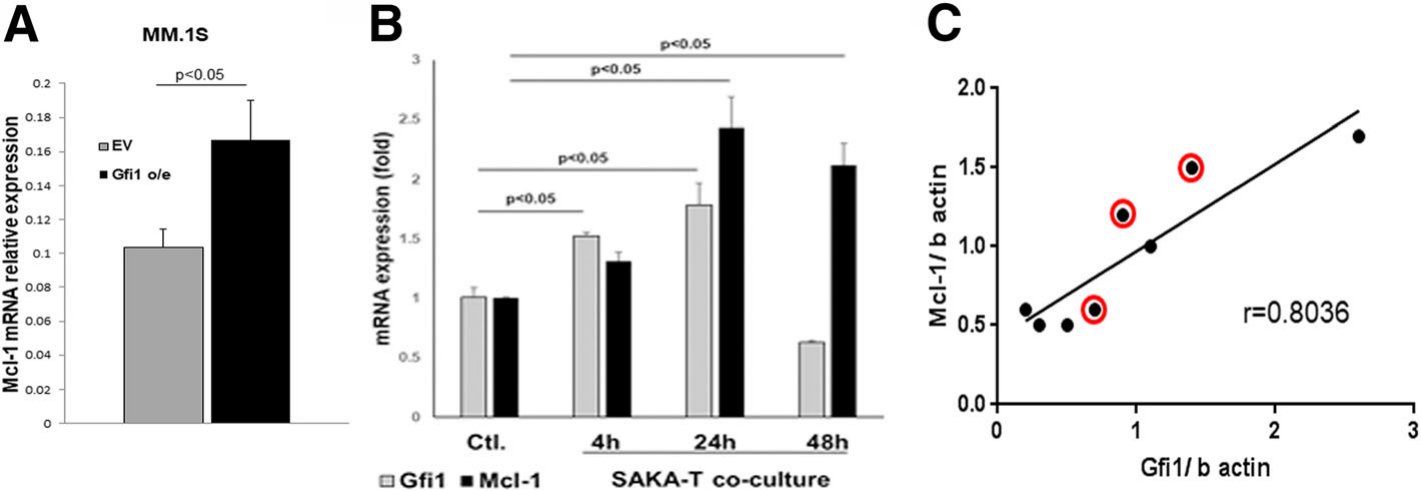
Gfi1 expression correlates with Mcl-1 expression in MM cells. *Mcl-1* mRNA levels were evaluated by qPCR in MM.1S stably overexpressing Gfi1 and their respective controls EV (**a**). H929 cells were directly co-cultured for 4, 24, and 48 h with the normal marrow stromal cells (SAKA-T). The *Gfi1* and *Mcl-1* mRNA levels were analyzed by qPCR and expressed as fold change vs untreated MM cells (*p < 0.05 vs untreated control) (**b**). Direct correlation between Gfi1 and Mcl-1 protein levels in myeloma cells (five MM cell lines and CD138+ cells from three MM patients marked with red circles) as analyzed by WB in Fig. 1d (**c**)

Gfi1 levels are elevated in early B cell development and decrease in mature B cells [8], but how Gfi1 expression is regulated in MM cells is unknown. Since multiple factors increase MM cell survival, growth, and chemoresistance [28], including adhesive interactions between MM cells and BMSC, IL-6, TNFα, and sphingosine-1-phosphate (S1P), we tested their effects on Gfi1 expression in MM cells. Adhesive interactions between MM cells and BMSC increased Gfi1 expression 1.5-fold in MM cells, at both the transcriptional and protein level (Fig. 5a) as did IL-6 (Fig. 5b). S1P and TNFα had variable effects on Gfi1 levels in MM cell lines (Fig. 5b). Both IL-6 treatment and MM-BMSC adhesive interactions significantly enhanced Gfi1 protein levels, which were associated with increased levels of the pro-survival Mcl-1 protein levels in MM cells (Fig. 5a, right panel; c; d). Since *Mcl-1* is a direct and functional target gene of Gfi1 in p210BCR/ABL-transformed cells [29] and plays an important role in cell proliferation and survival, we tested if Gfi1 and Mcl-1 protein expression levels were correlated in MM cells. *Mcl-1* mRNA levels were significantly increased in MM.1S *Gfi1* o/e cells compared with control MM.1S EV cells (Fig. 6a). Further, adhesive interactions with BMSC (SAKA-T normal human BMSC cell line) enhanced *Gfi1* mRNA expression in H929 MM cells as early as 4 h, and this induction positively correlated with the increased *Mcl-1* expression (Fig. 6b). Importantly, Gfi1 protein levels in MM cell lines and primary CD138+ MM cells significantly and highly correlated with Mcl-1 protein expression (Fig. 6c). Thus, BM microenvironmental factors known to sustain MM cell growth and survival also regulate Gfi1.

### Overexpression of Gfi1 in MM cells increases MM tumor burden and osteolysis in vivo

**Fig. 7:**
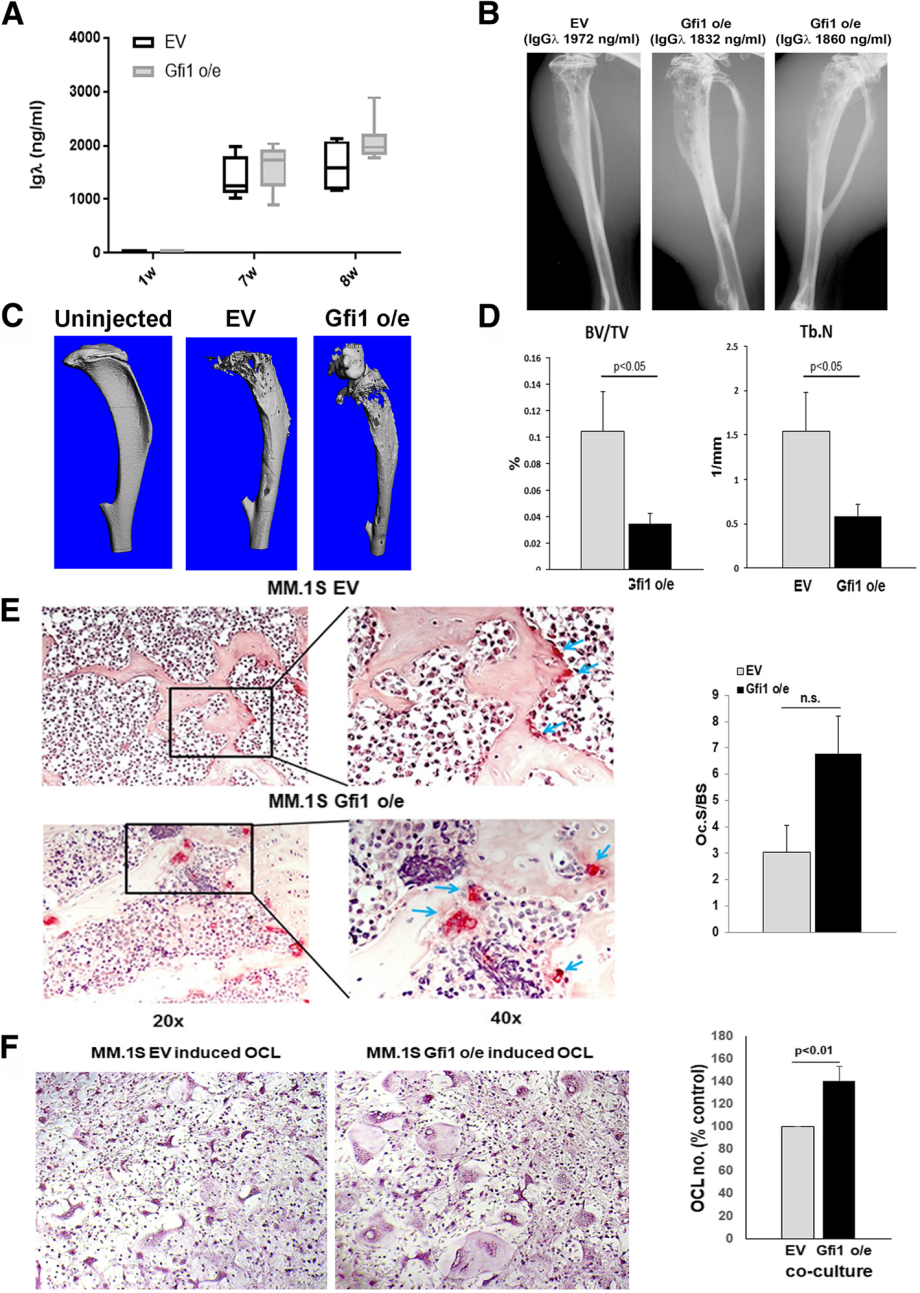
Gfi1 overexpression in MM.1S enhances bone loss in the MM-bearing mice and MM-induced OCL formation. Plasma Igλ concentrations were measured by ELISA, 1 week (1w) and 7 weeks (7w) after the intratibial injection of MM cells and at the time of harvest (8w) in both MM.1S Gfi1 o/e- (*N* = 7) and EV-injected mice (*N* = 6) (**a**). X-ray of three tibiae from an EV and two Gfi1 o/e-injected animals displaying similar plasma Igλ levels (**b**). Fixed tibiae were scanned using a μCT system (vivaCT 40, Scanco Medical). Representative μCT images demonstrating osteocytic lesions in the tibias of MM.1S Gfi1 o/e- and MM.1S EV-bearing mice compared with uninjected tibia (**c**). The bone tissue was measured by relative volume of calcified tissue (BV/TV) (*N* = 7) and the trabecular number (Tb.N.) (*N* = 6) in mice injected IT with (10^5^) Gfi1 o/e or EV MM.1S cells, respectively. Data are shown as mean ± SEM (**d**). Representative microscopic images of bone sections stained with TRAP to identify osteoclasts (arrows) on cancellous bone of MM.1S Gfi1 o/e- and EV-bearing mice. Magnification × 20 (on the left) and representative areas (black squares) are magnified on the right (× 40). The bar graph on the right shows OCL surface normalized to bone surface (Oc.S/BS) from MM.1S Gfi1 o/e- (*N* = 6) and EV-bearing mice (*N* = 4). Data are shown as mean ± SEM (**e**). Mouse OCL precursors, obtained from murine bone marrow after stimulation with MCSF (10 ng/ml) and RANKL (50 ng/ml), were co-cultured with MM.1S EV and MM.1S Gfi1 o/e myeloma cells for 72 h and then TRAP stained. The bar graph represents the OCL number expressed as percentages normalized to EV controls. Values are mean ± SD; *p < 0.05. Representative microscopic images of TRAP-positive OCL after 72 h exposure to either MM.1S EV (left panel) or MM.1S Gfi1 o/e (right panel) showing increase number and size of OCL in cultures exposed to MM.1S Gfi1 o/e MM cells (**f**)

We next determined the effects of *Gfi1* overexpression in MM cells in vivo. Consistent with prior studies, 80% of evaluable animals injected intratibially with either MM.1S EV cells (6/7) or MM.1S Gfi1 o/e cells (7/9) successful engrafted MM cells, with detectable human Igλ levels in plasma as an estimate of tumor burden. Plasma Igλ concentrations at sacrifice were higher in MM.1S *Gfi1* o/e-injected mice as compared with MM.1S EV-injected mice, although this enhanced MM growth did not reach statistical significance (Fig. 7a). Histologic analysis of tumor burden in MM bearing tibiae was difficult to assess due to extensive cortical bone destruction in bones injected with the MM.1S *Gfi1* o/e cells as compared with MM.1S EV-injected mice (data not shown). The trend towards more aggressive tumors in the bone marrow of mice bearing *Gfi1* o/e MM.1S as compared with EV controls might be due to the modulation of the c-Myc oncogene by the Gfi1 levels (Additional file 5: Figure S3A), Interestingly, examination of tibial X-rays of animals displaying similar Igλ showed that MM.1S Gfi1 o/e cells caused greater and more extensive cortical and trabecular bone destruction than MM.1S EV cells (Fig. 7b). μCT analysis confirmed the significantly greater bone loss in the injected tibiae of MM.1S Gfi1 o/e-bearing mice (Fig. 7c) with a significant decrease in bone volume relative to the volume of calcified tissue (BV/TV) and trabecular number (Tb.N.) (Fig. 7d). Importantly, histologic analysis of tartrate-resistant acid phosphatase (TRAP)-stained sections showed increased osteoclast (OCL) numbers in the Gfi1 o/e MM.1S-bearing tibias compared with EV MM.1S-bearing tibias (Fig. 7e, histology). Analysis of OCL surface area (Oc.S/BS) demonstrated a marked increase in the Gfi1 o/e MM.1S-injected tibias that did not reach statistical significance (Fig. 7e, bar graph), again reflecting the extreme bone loss seen in these animals. Interestingly, the histologic analysis with TRAP staining demonstrated that the Gfi1 o/e MM.1S-injected tibias had larger OCL that contained more nuclei/OCL (Fig. 7e, histology). We then determined if increased Gfi1 expression in the MM cells increased OCL formation by culturing purified mouse OCL precursors with Gfi1 o/e and EV MM.1S cells for 72 h. TRAP staining of the cultures showed significantly increased OCL numbers (*p* < 0.05) in co-cultures containing Gfi1-overexpressing MM cells (Fig. 7f). Further, OCL formed in co-cultures of Gfi1 o/e MM.1S were larger and contained more nuclei/OCL than OCL formed in EV MM.1S control cell co-cultures. Consistent with this findings, in preliminary studies, we found that MM.1S Gfi1 o/e cells produce higher protein levels of IL-6 and integrin α4 as well as mRNA levels of RANKL and IL-6 and secrete higher levels of MIP-1α than MM.1S EV controls (Additional file 5: Figure S3 B, C, and D). These results support that Gfi1 o/e in MM cells enhances OCL precursor fusion and OCL formation.

## Discussion

Gfi1 is a proto-oncoprotein [13] that acts as a transcriptional repressor, which can regulate cell fate, differentiation, and survival in normal and malignant hematopoiesis [25, 30]. Previous studies showed that Gfi1 affected T cell survival by inhibiting apoptosis through repression of multiple pro-apoptotic regulators such as BAX and BAK [9] and that loss of Gfi1 impairs proliferation and survival of early myeloid cells [31]. However, Gfi1’s role in MM was previously unknown. We found that MM cell lines and CD138^+^ cells from MM patients expressed elevated levels of Gfi1 when compared to CD138^+^ cells from healthy donors. In addition, *Gfi1* gene expression levels in MM patient CD138+ cells correlated with disease progression, suggesting a potential role for Gfi1 in MM progression. This observation is consistent with previous studies that showed Gfi1 is involved in the accelerated progression of lymphoid malignancies in MoMuLV-infected mice [13, 32, 33] and that Gfi1 can act as an oncogene to enhance lymphomagenesis through cooperation with Myc and Pim-1 [13, 33, 34]. Furthermore, our observation is in line with a recent study showing that Gfi1 overexpression contributes to enhanced tumorigenesis in medulloblastoma [35] and small cell lung cancer [36].

We demonstrated that Gfi1 decreased MM cell death by inhibiting expression of apoptosis-inducing genes, increasing cell growth, and decreasing sensitivity of these cells to proteasome inhibitor-induced apoptosis. Further, loss of Gfi1 had profound pro-apoptotic effects on MM cells, increasing BAX, PUMA, and NOXA as well as cleaved caspase 3 protein levels in p53-replete cells and significantly decreased the proliferative capacity of MM cells. Importantly, *Gfi1 o/e*-granted MM cells had a proliferative advantage over EV-transfected controls, by enhancing the percentage of cells in the G2+M cell cycle phase. These results suggest an important role for Gfi1 in MM cell survival and growth [37, 38].

To determine if Gfi1 could contribute to drug resistance in MM, we assessed its capacity to block the effect of Btz and dexamethasone-induced apoptosis, major components of MM therapy [39, 40]. We found that blocking proteasomal degradation with low concentrations of Btz (3 nM) consistently activated caspase 3 cleavage in MM cells, but this was accompanied by increased Gfi1 protein levels. Higher doses of Btz (5 nM) induced a dramatic enhancement of apoptosis in MM cells that was associated with the loss of the increased levels of Gfi1. Importantly, in the MM cells overexpressing Gfi1, Gfi1 protein accumulation persisted, regardless of the Btz concentrations. The increased expression of Gfi1 conferred protection of MM cells to Btz-induced apoptosis, as shown by the low levels of active caspase 3 and the significantly higher cell viability. The twofold overexpression of *Gfi1* did not confer any viability advantage to MM cells treated with dexamethasone. Given previous reports showing that Gfi1 expression is regulated at the protein level through ubiquitin-proteasome-mediated degradation [14, 41], our results suggest that Gfi1 may contribute to Btz-induced drug resistance in MM cells, which may, in part, result from persistent Gfi1 accumulation.

We found that ablation of Gfi1 leads to MM cell death through induction of p53-dependent pro-apoptotic proteins in p53-wt MM cell lines. Moreover, we found that Gfi1 binds to p53, preventing its binding to the BAX and NOXA promoters and that Gfi1-p53 binding was blocked by acetylation of Gfi1. The interaction between Gfi1 and p53 has been described previously in other systems. In T-ALL, Khandanpour et al. [12] demonstrated that Gfi1 recruits LSD1 to p53 and dampens its activity by de-methylating p53 at C-terminal lysines to prevent immediate apoptosis. Du and collaborators reported that p53 represses transcription of Gfi1 in human lymphoma cells [17], whereas Liu and colleagues found that Gfi1 is a positive p53 target in hematopoietic cells [42]. However, Gfi1-p53 interactions have not been described in MM cells. Our results clearly show that Gfi1-p53 interactions occur in MM cells and promote MM cell survival by preventing p53 binding to promoters of pro-apoptotic target genes (*BAX*, *NOXA*). Moreover, we show for the first time that Gfi1 is a protein whose function can be modulated by acetylation. Using truncated Gfi1 constructs, we found an acetylation site between Gfi1 residues 291–341 that was necessary for Gfi1-p53 binding, as shown by our studies with the Gfi1 K292R mutant. Cell fractionation studies showed that under basal conditions, Gfi1 and p53 are primarily localized in the cytosol of MM cells. Acetylation induced by HDACi treatment decreased the amount of total p53 in the cytosol and increased its acetylated form in the nucleus, while the nuclear-cytosolic distribution of Gfi1 remained unchanged. These results suggest that acetylation of Gfi1 is a critical step in p53’s translocation to the nucleus to induce apoptosis in MM cells.

Our results suggest that Gfi1 also plays a key role in the survival and growth of p53-mutant or p53-null MM cells. Although p53 mutations are rare in MM, chromosome 17p13 deletions are detectable in about 10% of newly diagnosed patients [43]. Patients harboring the 17p13 deletion [del(17p)] are considered “high risk” and have poorer outcomes and shorter survival times compared to standard-risk patients [44–46]. Moreover, the prevalence of [del(17p)] increases in advanced stages of disease such as plasma cell leukemia and extramedullary disease [44].

The mechanism by which Gfi1 regulates the survival and growth of these cells remains unknown. As reported in T cells, one possibility is that in p53-mutant or p53-null MM cells, Gfi1 interacts directly with ETS1 to repress the *BAX* gene through adjacent DNA binding sites [47] or that Gfi-1 interacts with PIAS3 to relieve its inhibitory effect on STAT3 activity [48]. Further studies are required to determine if these occur, or if other mechanisms are involved in MM.

The MM microenvironment plays a critical role in MM cell survival and growth. High levels of IL-6 are produced by BMSC and increase the growth and survival of normal B cell lineage and MM cells [49]. Further, adhesive interactions between BMSC and MM cells enhance MM cell growth and drug resistance [50]. We found that IL-6 and adhesive interactions with BMSC cells consistently upregulated Gfi1 in MM cells at the transcriptional and protein levels. Moreover, Gfi1 protein levels were highly correlated with Mcl-1 protein levels. Mcl-1 is required for proliferation and survival of hematopoietic stem cells [51, 52] and is a transcriptional target of Gfi1 in chronic myelogenous leukemia [29].

Most importantly, our in vivo study showed that mice bearing *Gfi1* o/e MM.1S cells developed more aggressive tumors in the bone marrow as compared with EV controls, although the tumor burden was difficult to assay histologically because of its extramedullary growth. The difference in the oncogenic phenotype might be due to the modulation of the c-Myc oncogene by the Gfi1 levels (Additional file 5: Figure S3A), since c-Myc overexpression was been related to poor prognosis in MM patients [53]. Mice bearing Gfi1 o/e MM tumors had more bone destruction than those bearing control MM cells and larger OCLs with more nuclei/cell. Hypernucleated OCL have an increased bone resorbing capacity [54]. Further, Gfi1 o/e MM cells enhanced OCL precursor fusion and OCL formation in vitro. The underlying mechanism for the OCL effect is beyond the scope of this study but may involve increased secretion of several soluble osteoclastogenic factors by MM cells such as MIP1α, RANKL, or MMP13 [54].

## Conclusions

In summary, our results support Gfi1 as a key contributor to MM cell survival and growth through its regulation of p53 activity in p53-replete MM cells and that Gfi1 can be targeted in p53-replete MM cell by HDACi treatment. Further, our results suggest that Gfi1 may contribute to Btz resistance and that targeting Gfi1 may be a novel therapeutic strategy for MM patients, even those harboring p53 mutations or deletions.

## Additional files

Additional file 1: **Table S1.** Main characteristics of the US Patients and Normal donors cohort. **Table S2.** Main characteristics of the Italian patient’s cohort. **Table S3.** Sequences of qPCR primers used for amplification of human mRNA. (DOCX 22 kb)

Additional file 2: Chemicals and antibodies; Plasmid and lentiviral constructs; Immunoprecipitation and Western blotting; qPCR; ChIP assay; Immunofluorescence. (DOCX 22 kb)

Additional file 3: **Figure S1.** *Gfi1-*KD induces apoptosis in MM cells regardless of their p53 status. MM.1S cells, lentiviral infected to knock down *Gfi1* (Gfi1-shRNA #1) and the corresponding scrambled control (Scr-shRNA) were stained with PI and cell cycle phases were evaluated by flow cytometry. The histograms show the different amplitude of the “sub Go” phases representing different levels of apoptosis (A). *Gfi1* KD was induced by lentiviral infection (Gfi1-shRNA #1) in H929 cells (p53 wt), JJN3 (p53 haploinsufficient) and RPMI-8266 (p53 mutant) MM cell lines. Proteins collected 24 h after the puromycin selection were analyzed by WB for pro-apoptotic cleavage of Mcl-1 (Mcl-1(s)) and caspase 3 as compared to control lentiviral infected cells (Scr-shRNA) (B). (JPG 623 kb)

Additional file 4: **Figure S2.** *Gfi1* overexpression increases metabolic activity and confers protection from Btz-induced apoptosis in JJN3 MM cells. Stable cumate inducible Gfi1 (iGfi1) JJN3 cells and their respective controls (iCtl) were obtained as described in the Methods section. Gfi1 overexpression (4–5 fold compared to iCtl) (data not shown) was induced by exposing the cells to 25 μg/ml cumate for 24 h (overexpression was stable for 48 h after removing the cumate from culture media). MTT assays showing metabolic activity of JJN3 iGfi1 cells as compared with iCtl at 24 h after cumate was removed from the media (*N* = 4) (A). MTT assay showing metabolic activity of JJN3 iGf1 and iCtl cells, treated for 24 h and 48 h with Btz (3, 5 and 10 nM). The bar graph represents % versus untreated control (B). (JPG 262 kb)

Additional file 5: **Figure S3.** MM *Gfi1* o/e cells produce higher levels of osteoclastogenic factors. MM.1S EV and Gfi1 o/e cells (upper left panel; graph on the right represents densitometric evaluation of three independent experiments) and H929 *Gfi1-* shRNA and Scr-shRNA cells (lower left panel; graph on the right represents densitometric evaluation of three independent experiments) were analyzed by WB for Gfi1 and c-Myc protein expression using β-actin and α-tubulin as loading controls (A); MM.1S EV and Gfi1 o/e cells protein lysates were analyzed by WB for Gfi1, Integrin α4 and IL6 protein levels using GAPDH as loading control (B); RANKL and IL6 mRNA levels were measured by qPCR using specific primers in MM.1S EV and Gfi1 o/e cells (C); MIP1α protein levels were measured by ELISA (R&D Systems, Minneapolis, MN) in 72 h condition media harvested from MM.1S EV and Gfi1 o/e cells (D). (JPG 523 kb)

## Abbreviations

BM: Bone marrow
BMSC: Bone marrow stromal cell
Btz: Bortezomib
BV: Bone volume
ChIP: Chromatin immunoprecipitation
EDTA: Ethylenediaminetetraacetic acid
EV: Cells expressing empty vector
Gfi1 o/e: Cells overexpressing *Gfi1*
Gfi1: Growth factor independence 1
HDACi: Histone deacetylases inhibitors
IL-6: Interleukin 6
IP: Immunoprecipitation
ishRNA: Inducible short hairpin RNA
IT: Intratibial
KD: Knockdown
M-CSF: Macrophage colony-stimulating factor
MM: Multiple myeloma
NAM: NAD+-dependent HDAC inhibitor
OB: Osteoblast
OCL: Osteoclast
PI: Propidium iodide
qRT-PCR: Quantitative real-time PCR
RANKL: Receptor activator of nuclear factor kappa-Β ligand
S1P: Sphingosine 1 phosphate
Scr: Scramble
shRNA: Short hairpin RNA
T-ALL: T cell acute leukemia
TbN: Trabecular number
TNFα: Tumor necrosis factor α
TRAP: Tartrate-resistant alkaline phosphatase
TSA: Zn^2+^-dependent HDAC inhibitor
TV: Volume of calcified tissue
WB: Western blot
wt: Wild-type
