# Supplementary material for: Growth factor independence 1 expression in myeloma cells enhances their growth, survival, and osteoclastogenesis
Source: J Hematol Oncol. 2018 Oct 4;11:123. doi: 10.1186/s13045-018-0666-5 (PMC6172782; doi:10.1186/s13045-018-0666-5)
Supplement: Supplementary file 1 — Table S1. Main characteristics of the US Patients and Normal donors cohort. Table S2. Main characteristics of the Italian patient’s cohort. Table S3. Sequences of qPCR primers used for amplification of human mRNA. (DOCX 22 kb) [file 13045_2018_666_MOESM1_ESM.docx]

**Table S1: Main characteristics of the US Patients and Normal donors cohort**

| ***Patient*** | ***Clinical features*** | | | | | |
| --- | --- | --- | --- | --- | --- | --- |
| ***ID*** | ***Age (years)*** | ***Gender*** | ***Race*** | ***Newly diagnosed*** | ***ISS Stage*** | ***Skeletal disease*** |
| MM 1 | 55 | M | white | N | III | Y |
| MM 2 | 60 | F | white | N | III | Y |
| MM 3 | 73 | M | white | N | I | N |
| MM 4 | 79 | M | white | N | I | N |
| MM 5 | 55 | M | white | N | III | Y |
| MM 6 | 71 | F | white | N | I | Y |
| MM 7 | 60 | F | white | N | III | Y |
| MM 8 | 75 | M | white | N | I | N |
| MM 9 | 67 | M | white | Y | - | Y |
| MM 10 | 55 | F | white | Y | - | Y |
| MM 11 | 67 | F | white | Y | II | Y |

| ***Normal Donor***  ***ID*** | ***Age (years)*** | ***Gender*** | ***Race*** |
| --- | --- | --- | --- |
| ND 1 | 33 | F | white |
| ND 2 | 21 | F | white |
| ND 3 | 23 | M | white |
| ND 4 | 31 | M | white |
| ND 5 | 21 | M | white |
| ND 6 | 23 | F | white |

Abbreviations: MM- Multiple Myeloma; ND- normal donor.

**Table S2: Main characteristics of the Italian patient’s cohort**

| ***Patient*** | ***Clinical features*** | | | | | |
| --- | --- | --- | --- | --- | --- | --- |
| ***ID*** | ***Age (years)*** | ***Gender*** | ***Race*** | ***Newly diagnosed*** | ***ISS Stage*** | ***Skeletal disease*** |
| MGUS _I_1 | 44 | M | white | MGUS |  | N |
| MGUS _I_ 2 | 72 | F | white | MGUS |  | N |
| MGUS _I_ 3 | 72 | M | white | MGUS |  | N |
| MGUS _I_ 4 | 67 | F | white | MGUS |  | N |
| MMD_I_ 1 | \| 55 \| \| --- \| | M | white | Y | II | Y |
| MMD_I_ 2 | 66 | M | white | Y | III | Y |
| MMD_I_ 3 | 64 | F | white | Y | II | Y |
| MMD_I_ 4 | 81 | F | white | Y | III | N |
| MMD_I_ 5 | 57 | M | white | Y | I | Y |
| MMD_I_ 6 | 57 | M | white | Y | III | Y |
| MMD_I_ 7 | 83 | F | white | Y | II | Y |
| MMD_I_ 8 | 85 | M | white | Y | III | N |
| MMD_I_ 9 | 73 | M | white | Y | II | Y |
| MMD_I_ 10 | 75 | F | white | Y | II | Y |
| MMD_I_ 11 | 78 | M | white | Y | III | N |
| MMR_I_ 1 | 61 | F | white | N | III | Y |
| MMR_I_ 2 | 80 | F | white | N | I | Y |
| MMR_I_ 3 | 43 | M | white | N | I | Y |
| MMR_I_ 4 | 76 | M | white | N | III | Y |
| MMR_I_ 5 | 54 | F | black | N | I | ND |
| MMR_I_ 6 | 71 | F | white | N | II | Y |
| MMR_I_ 7 | 81 | F | white | N | III | Y |
| MMR_I_ 8 | 61 | F | white | N | III | Y |
| MMR_I_ 9 | 64 | M | white | N | II | N |
| MMR_I_ 10 | 73 | F | white | N | ND | Y |

Abbreviations: MGUS-Monoclonal Gammopathy of Undetermined Significance; MMD- Multiple Myeloma newly Diagnosed; MMR- Multiple Myeloma Relapsed, ND- not determined.

**Table S3: Sequences of qPCR primers used for amplification of human mRNA**

| **qPCR** | **Gene** | **Primer** | **Sequence 5’-3’** |
| --- | --- | --- | --- |
|  | **Gfi1** | Forward | **GAGCCTGGAGCAGCACAAAG** |
|  |  | Reverse | **GTGGATGACCTCTTGAAGCTCTTC** |
|  | **BAX** | Forward | **CCCCGAGAGGTCTTTTTCCG** |
|  |  | Reverse | **GGCGTCCCAAAGTAGGAGA** |
|  | **PUMA** | Forward | **GACCTCAACGCACAGTACGAG** |
|  |  | Reverse | **AGGAGTCCCATGATGAGATTGT** |
|  | **NOXA** | Forward | **ACCAAGCCGGATTTGCGATT** |
|  |  | Reverse | **ACTTGCACTTGTTCCTCGTGG** |
|  | **Mcl-1** | Forward | **TAAGGACAAAACGGGACTGG** |
|  |  | Reverse | **ACCAGCTCCTACTCCAGCAA** |
|  | **18s** | Forward | **ATC CCT GAA AAG TTC CAG CA** |
|  |  | Reverse | **CCC TCT TGG TGA GGT CAA TG** |
|  | | | |
| **ChIP** | **Gene** | **Primer** | **Sequence 5’-3’** |
|  | **BAX** | Forward | **GGGTTATCTCTTGGGCTCACAA** |
|  |  | Reverse | **GAGCTCTCCCCAGCGCA** |
|  | **NOXA** | Forward | **CTCGAGACCTGCTCCACTTC** |
|  |  | Reverse | **CGCTGGAATCCTCTCTGTTC** |
