# Supplementary material for: Growth factor independence 1 expression in myeloma cells enhances their growth, survival, and osteoclastogenesis
Source: J Hematol Oncol. 2018 Oct 4;11:123. doi: 10.1186/s13045-018-0666-5 (PMC6172782; doi:10.1186/s13045-018-0666-5)
Supplement: Supplementary file 2 — Chemicals and antibodies; Plasmid and lentiviral constructs; Immunoprecipitation and Western blotting; qPCR; ChIP assay; Immunofluorescence. (DOCX 22 kb) [file 13045_2018_666_MOESM2_ESM.docx]

**Additional file 2**

**Chemicals and antibodies**

Cell culture media, penicillin and streptomycin, CellTrace™ Far Red Proliferation Kit were from Invitrogen (Eugene, OR). Recombinant human IL-6, TNF-α were from R&D Systems and Bortezomib (Btz) was from Selleck chemicals (Houston, TX). Anti-Gfi1, anti-BAX, anti-PUMA, anti-NOXA and anti-GAPDH antibodies were from Santa Cruz Biotechnology (Dallas, TX). Anti-c-Myc, anti-acetylated-Lysine, anti-Caspase 3 antibodies were from Cell Signaling Technology (Beverly, MA) and anti-Mcl-1 antibodies were from Abcam (Cambridge, MA). Anti-β-actin and anti-α-tubulin antibodies as well as Propidium iodide, Trichostatin A and nicotinamide were from Sigma-Aldrich (Saint Louis, MO). Sphingosine-1-phosphate (S1P) was from Cayman Chemical (Ann Arbor, MI). HRP-conjugated anti-rabbit and anti-mouse antibodies were from Life Technologies (Gaithersburg, MD).

**Plasmid and lentiviral constructs**

For Gfi1 acetylation site studies, murine (m)Gfi1 cDNA constructs in full length (pcDNA3.1-Myc-mGfi1wt) or truncated forms (pcDNA3.1-Myc-mGfi1(1-261); pcDNA3.1-Myc-mGfi1(1-300); pcDNA3.1-Myc-mGfi1(239-423); pcDNA3.1-Myc-mGfi1(291-423); pcDNA3.1-Myc-mGfi1(341-423); pcDNA3.1-Myc-mGfi1(1-423)) were used to express Gfi1 protein in HEK293-T cells by transient transfections using Lipofectamine 2000 (Invitrogen, Eugene, OR). Site-directed mutagenesis was employed to change mGfi1 lysine 292 to arginine (pcDNA3.1-Myc-mGfi1-K292R) using QuikChange II Site-Directed Mutagenesis Kit (Agilent Technologies, Santa Clara, CA). For Gfi1-p53 binding studies HEK293-T cells were co-transfected with pcDNA3.1-HA-p300 (containing the common lysine acetyltransferase) and the Gfi1 containing plasmids. Mouse and human Gfi1 full length cDNA sequence are highly homologous and HEK293-T cells have undetectable levels of Gfi1 protein, making them a suitable system to study the interactions between the exogenous Gfi1 deletion constructs and endogenous p53. *Gfi1* KD in MM cells was generated by transient transduction with pLKO.1-puro lentivirus encoding Gfi1 (two different validated shRNA constructs were used) or non-mammalian shRNA (MISSION shRNA form Sigma-Aldrich, St. Louis, MO, USA) followed by 48h puromycin selection. The MM.1S cells, stable overexpressing *Gfi1* (*Gfi1* o/e) were obtained by transduction with puc2CL6IEGwo-GFP lentivirus encoding Gfi1 or the empty vector (EV) after selection by GFP flow-cytometry using a BD FACSAria II sorter (BD Bioscience, San Jose, CA). The stable inducible *Gfi1* o/e JJN3 cells were obtained by co-transduction with iCumate-pLenti-Gfi1-SV40-GFP (containing Gfi1 sequence) or empty vector and iCumate-pLenti-EF1a-CymR-Neo (containing the CymR repressor that binds the cumate operator sequence) from Applied Biological Materials (ABM) Inc. (Richmond BC, Canada) followed by selection with puromycin and neomycin.

**Immunoprecipitation and Western blotting**

Cell pellets were lysed with IP lysis buffer containing 0.01% SDS, 1% Triton X-100, 50 mM Tris-HCl (pH7.5), 150 mM NaCl, 0.5 mM EDTA, 1 mM DTT, 0.1 mM Na3VO4, 0.1 mM Na_4_P_2_O_7_, 0.1 mM NaF, and 1% proteinase inhibitor cocktail (Sigma, P8340). 500 μg of lysates were pre-cleared using protein G agarose (Millipore, Temecula, CA, Cat. No. 16-266) for 1 h and incubated with control IgG, anti-Myc or anti-Gfi1 antibodies overnight at 4°C. Protein/antibody complexes were precipitated by protein G agarose for 2 h. Denatured protein complexes were separated by 10% SDS-PAGE gel electrophoresis and transferred to PVDF membrane. For regular western blotting, protein lysates were extracted in RIPA lysis buffer (sc-24948; SCB) supplemented with protease inhibitors cocktail (Sigma-Aldrich, Saint Louis, MO). Equal amounts of proteins, as determined by bicinchoninic acid assay protein analysis (Pierce, Rockford, IL), were separated on Any kD^TM^ SDS-PAGE gels (Bio-Rad Laboratories, Hecules, CA) and transferred onto PVDF membranes. For the detection of immune complexes, the membranes were incubated with various primary antibodies and specific HRP-linked secondary antibodies, which were detected using the enhanced chemiluminescence kit (Thermo Scientific, Rockford, IL). The immune complexes were quantified by densitometry using ImageJ software after normalization to specific loading controls.

**Real-time RT-PCR (qPCR)**

Total mRNA was extracted using RNeasy (QIAGEN, Germantown, MD ) per the manufacturer’s protocol and reverse-transcribed using High capacity cDNA reverse transcription kit (Applied Biosystem, Foster City, CA) on a T100 Thermal Cycler (Bio-Rad Laboratories, Hercules, CA). Quantitative PCR was performed on an CFX96 Real-Time System (Bio-Rad Laboratories, Hercules, CA) using a SsoAdvanced SYBR Green Supermix (Bio-Rad Laboratories, Hercules, CA) and cDNA equivalent to 40 ng RNA in a 10 μl reaction according to the manufacturer’s instructions. The DNA sequences of human primers used for qPCR are listed in Supplemental Table S3. Relative expression was calculated using the comparative 2^-ΔΔCt^ method, with 18S rRNA used as a housekeeping gene.

**ChIP assay**

Chromatin from H929 or MM.1S cells was analyzed using SimpleChIP ® kit (Cell Signaling Technology, Danvers, MA, US) per manufacturer protocol using Protein G Beads (Millipore,). In brief, a total of 10^7^ cells were fixed in 1% formaldehyde for 10 min at room temperature. The cells were lysed in 1% SDS lysis buffer containing 10 mM EDTA and 50 mM Tris (pH 8.1). The lysates were sonicated (to generate DNA fragments of 250 base pairs (bp) average length) on ice using a Fisher Scientific Sonic Dismembrator (Model CL-18) and centrifuged at 12000 RPM for 10 min. 10 μg of sheared DNA per each sample was incubated with anti-p53 overnight at 4°C and precipitated using Protein G magnetic beads for 2 h. Aliquots for input and non-specific IgG control samples were included with each experiment. Purified DNA was obtained using spin column after overnight treatment with 5M NaCl and Proteinase K at 65°C. Real time PCR was performed using ChIP-*q*PCR primers for BAX and NOXA (Supplemental Table S3).Fold enrichment was calculated based on Ct as 2^ΔCt^, where ΔCt = (Ct_Input_ – Ct_IP_).

**Immunofluorescence**

Air died cytospins containing H929 cells were fixed in 4% paraformaldehyde for 15 min, washed in PBS, and blocked with serum (5% donkey and 5% goat) for 30 min. Slides were incubated with rabbit anti-p53 and mouse anti-Gfi1 primary antibody (Santa Cruz, CA) in PBST containing 1% BSA and 0.3% Triton X-100 for 1 h. The PBS washed slides were then incubated with Donkey anti-Mouse IgG (H+L), Alexa Fluor® 488 and Goat anti-Rabbit IgG (H+L), Alexa Fluor® 594 for 1h. Slowfade Gold antifade (Invitrogen, Eugene, OR) containing DAPI (4, 6-diamidino-2-phenylindole) was used to mount the slides.
