# Supplementary figures and images for: Growth factor independence 1 expression in myeloma cells enhances their growth, survival, and osteoclastogenesis
Source: J Hematol Oncol. 2018 Oct 4;11:123. doi: 10.1186/s13045-018-0666-5 (PMC6172782; doi:10.1186/s13045-018-0666-5)

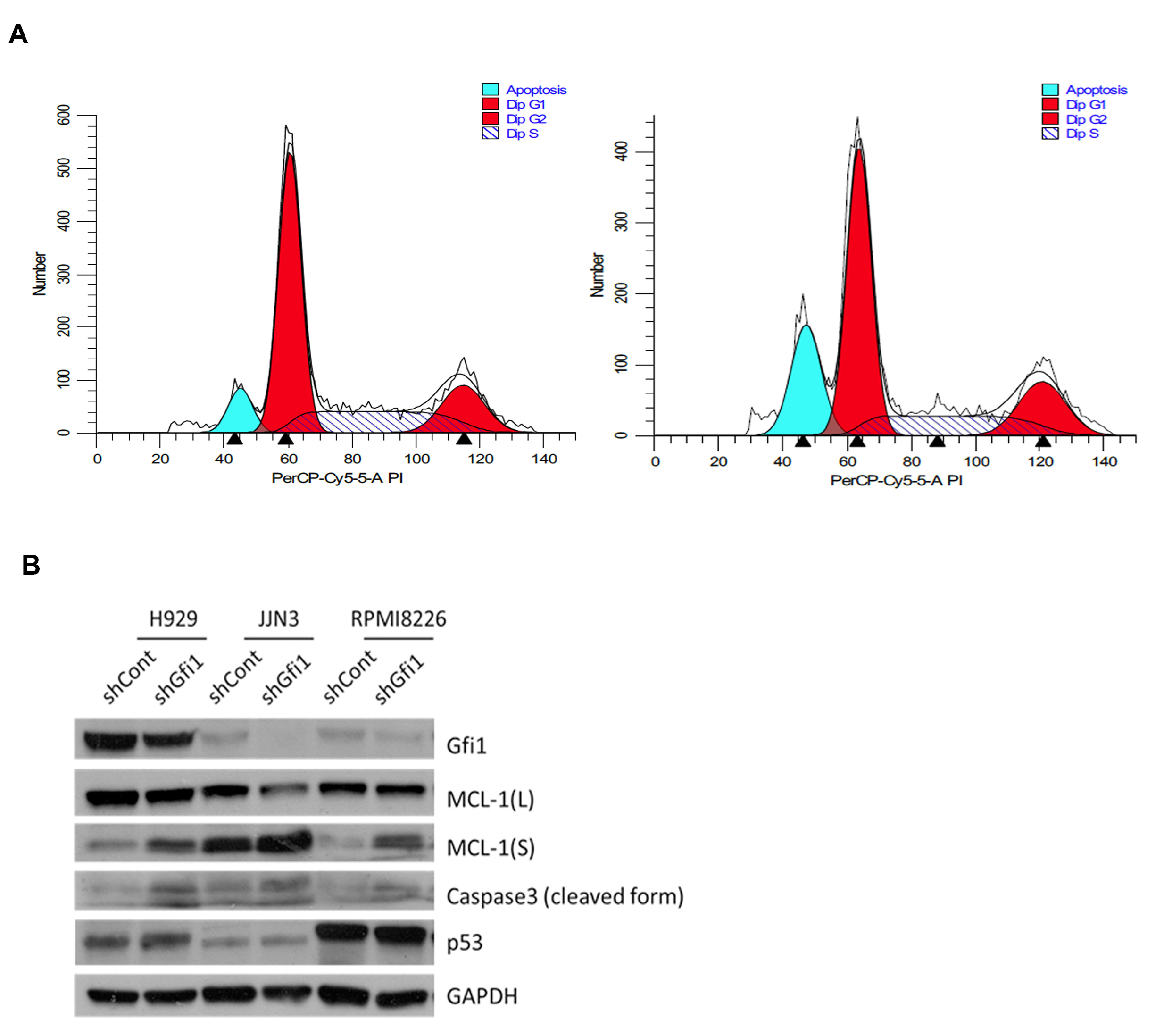

Supplement: Supplementary file 3 — Figure S1. Gfi1-KD induces apoptosis in MM cells regardless of their p53 status. MM.1S cells, lentiviral infected to knock down Gfi1 (Gfi1-shRNA #1) and the corresponding scrambled control (Scr-shRNA) were stained with PI and cell cycle phases were evaluated by flow cytometry. The histograms show the different amplitude of the “sub Go” phases representing different levels of apoptosis (A). Gfi1 KD was induced by lentiviral infection (Gfi1-shRNA #1) in H929 cells (p53 wt), JJN3 (p53 haploinsufficient) and RPMI-8266 (p53 mutant) MM cell lines. Proteins collected 24 h after the puromycin selection were analyzed by WB for pro-apoptotic cleavage of Mcl-1 (Mcl-1(s)) and caspase 3 as compared to control lentiviral infected cells (Scr-shRNA) (B). (JPG 623 kb) [file 13045_2018_666_MOESM3_ESM.jpg]

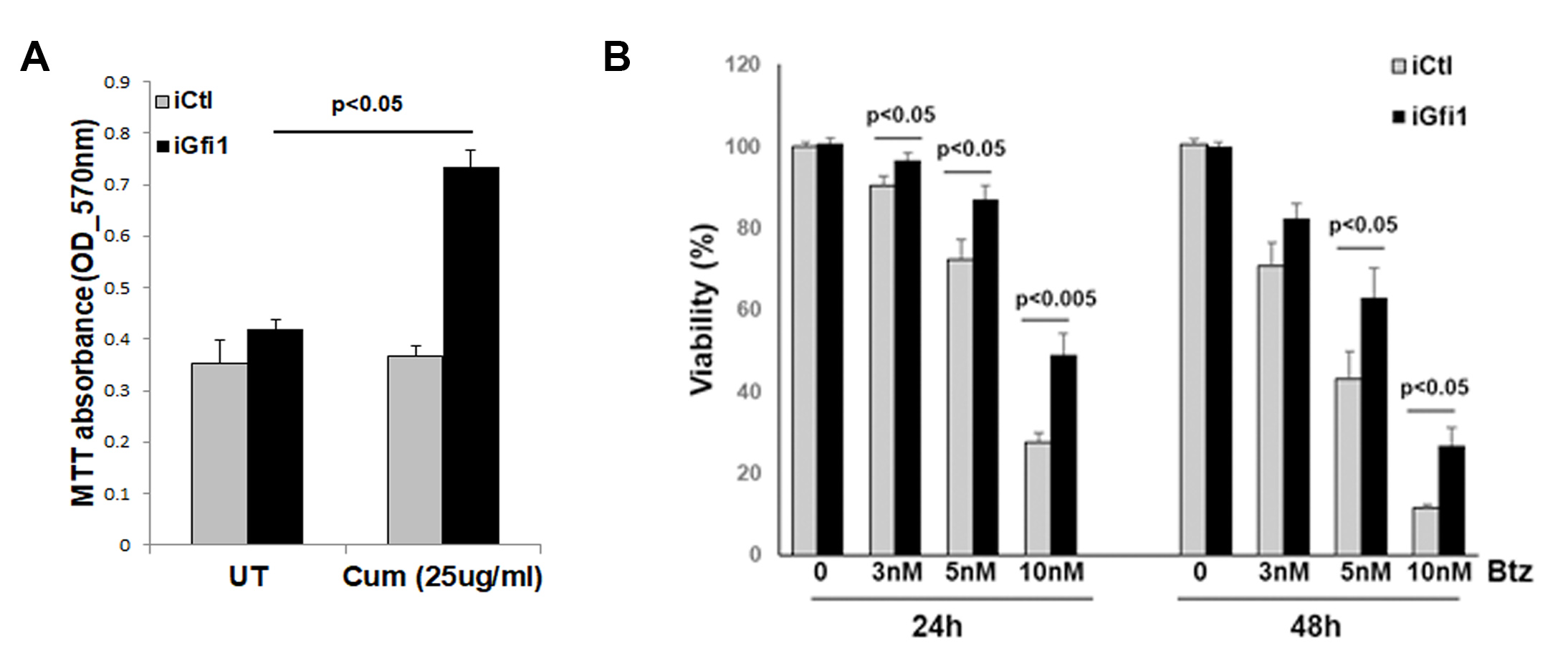

Supplement: Supplementary file 4 — Figure S2. Gfi1 overexpression increases metabolic activity and confers protection from Btz-induced apoptosis in JJN3 MM cells. Stable cumate inducible Gfi1 (iGfi1) JJN3 cells and their respective controls (iCtl) were obtained as described in the Methods section. Gfi1 overexpression (4–5 fold compared to iCtl) (data not shown) was induced by exposing the cells to 25 μg/ml cumate for 24 h (overexpression was stable for 48 h after removing the cumate from culture media). MTT assays showing metabolic activity of JJN3 iGfi1 cells as compared with iCtl at 24 h after cumate was removed from the media (N = 4) (A). MTT assay showing metabolic activity of JJN3 iGf1 and iCtl cells, treated for 24 h and 48 h with Btz (3, 5 and 10 nM). The bar graph represents % versus untreated control (B). (JPG 262 kb) [file 13045_2018_666_MOESM4_ESM.jpg]

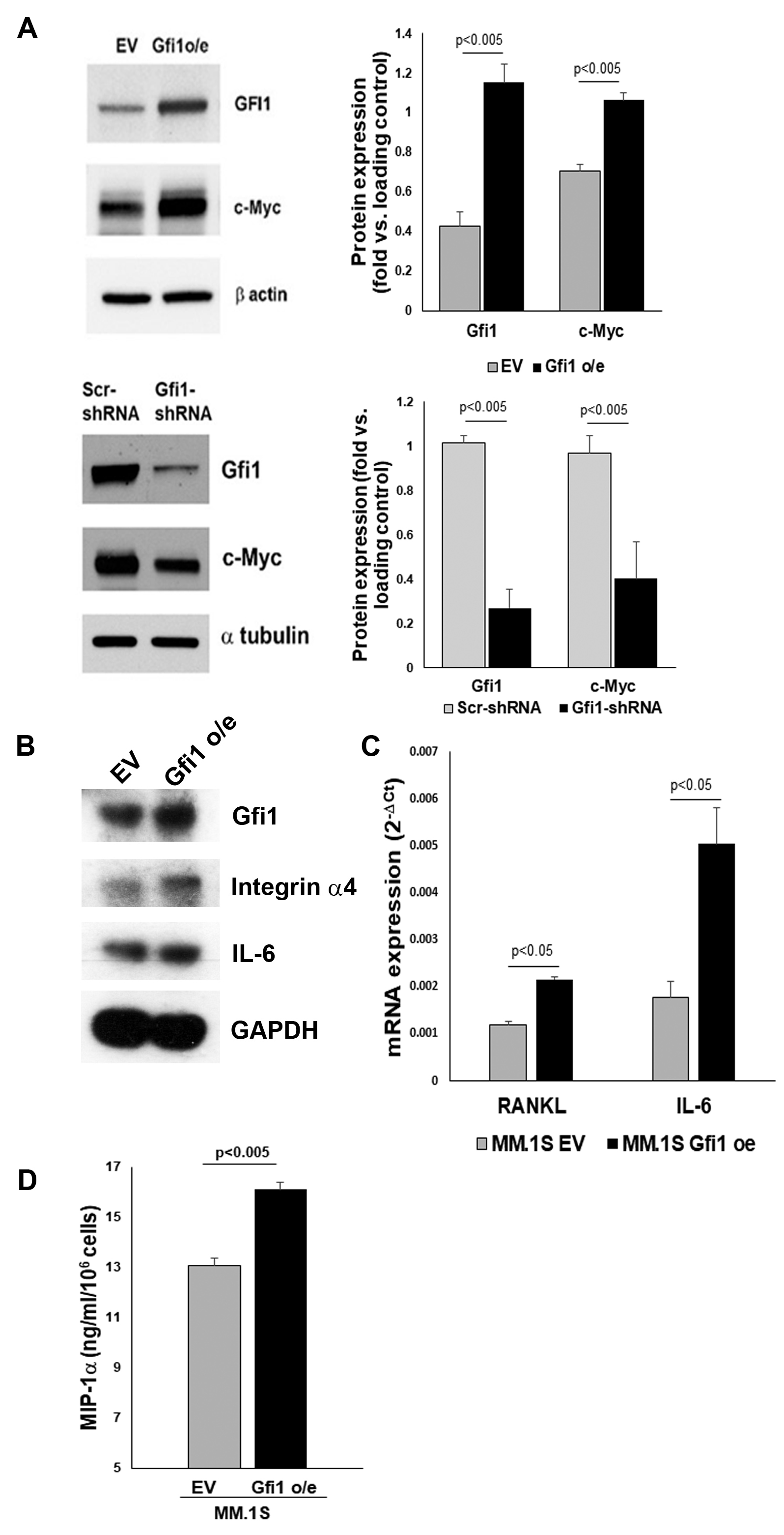

Supplement: Supplementary file 5 — Figure S3. MM Gfi1 o/e cells produce higher levels of osteoclastogenic factors. MM.1S EV and Gfi1 o/e cells (upper left panel; graph on the right represents densitometric evaluation of three independent experiments) and H929 Gfi1- shRNA and Scr-shRNA cells (lower left panel; graph on the right represents densitometric evaluation of three independent experiments) were analyzed by WB for Gfi1 and c-Myc protein expression using β-actin and α-tubulin as loading controls (A); MM.1S EV and Gfi1 o/e cells protein lysates were analyzed by WB for Gfi1, Integrin α4 and IL6 protein levels using GAPDH as loading control (B); RANKL and IL6 mRNA levels were measured by qPCR using specific primers in MM.1S EV and Gfi1 o/e cells (C); MIP1α protein levels were measured by ELISA (R&D Systems, Minneapolis, MN) in 72 h condition media harvested from MM.1S EV and Gfi1 o/e cells (D). (JPG 523 kb) [file 13045_2018_666_MOESM5_ESM.jpg]
